# Identification of exosomal circSLC26A4 as a liquid biopsy marker for cervical cancer

**DOI:** 10.1371/journal.pone.0305050

**Published:** 2024-06-11

**Authors:** Yutong Tong, Lanlan Jia, Minghui Li, Hongjuan Li, Shuli Wang

**Affiliations:** 1 Xinxiang Medical University, Xinxiang, Henan, P.R. China; 2 Department of Obstetrics and Gynecology, Zhengzhou Central Hospital Affiliated to Zhengzhou University, Zhengzhou, Henan, P.R. China; UFRN: Universidade Federal do Rio Grande do Norte, BRAZIL

## Abstract

**Objective:**

Circular RNA SLC26A4 (circSLC26A4) functions as an oncogene in the initiation and progression of cervical cancer (CC). However, the clinical role of plasma exosomal circSLC26A4 in CC is poorly known. This study aims to develop an accurate diagnostic method based on circulating exosomal circSLC26A4.

**Methods:**

In this study, exosomal circSLC26A4 derived from CC cell lines (CaSki, SiHa, and HeLa) and human cervical epithelial cells (HcerEpic) was measured and compared using quantitative reverse transcriptase polymerase chain reaction (qRT-PCR). Additionally, 56 volunteers, including 18 CC patients, 18 cervical high-grade squamous intraepithelial lesion (HSIL) patients, and 20 healthy volunteers, were enrolled. qRT-PCR was also performed to measure the plasma exosomal circSLC26A4 levels in all participants.

**Results:**

The exosomal circSLC26A4 expression level derived from CC cells was significantly elevated compared to it derived from HcerEpic cells. Plasma exosomal circSLC26A4 levels in CC patients were significantly higher than in healthy women and HSIL patients (*P* < 0.05). In addition, high plasma exosomal circSLC26A4 expression was positively associated with lymph node metastasis and FIGO stage (all *P* < 0.05). However, no significant correlation was found between plasma exosomal circSLC26A4 expression and age, intravascular cancerous embolus, and perineural invasion (*P* > 0.05).

**Conclusions:**

The high exosomal circSLC26A4 expression is closely related to the occurrence of CC. Plasma exosomal circSLC26A4 can be used as a diagnostic marker for CC.

## Introduction

In 2020, cervical cancer (CC) ranked as the fourth most prevalent cancer in women globally, with the worldwide occurrence of 13.3 cases per 100,000 individuals [1]. The incidence and mortality of CC are increasing yearly and are trending younger in China [2]. Colposcopy is an important link in CC screening [3]. Biopsy guided by colposcopy is widely used in diagnosing CC diagnosis, despite its inherent defects, such as invasiveness. Nevertheless, laser conization or loop electrosurgical excision procedure (LEEP) samples identified as many as 10% of micro-invasive or invasive cancers that were missed in targeted biopsies [4]. Hence, there is a pressing need for new and dependable indicators to identify and manage CC.

Exosomes are tiny vesicles with a of 40–160nm diameter that can be present in all body fluids and are becoming important in treating and diagnosing diseases [5]. Exosomes contain a wealth of genetic material, such as DNA, mRNA, microRNA, long noncoding RNA, and circular RNA, that are essential in the progression of cancer [6]. The circRNA is a circular structure formed by the reverse head-to-tail joining of exon gene sequences, which is widely present in eukaryotes as a reverse variable splicing product [7]. Recent studies have demonstrated that stable-structured circRNAs have emerged as a new star among tumor molecular markers, attracting the attention of international researchers. Exosomal circRNAs may also be useful biomarkers for cancer screening, diagnosis, and monitoring. Exosomal circ-PED8A, circNRIP1, and circPTGR1 are enriched in different cancer tissues, demonstrating that communication between cancer cells and the surrounding stroma plays a role in metastasis [8–10]. Various circRNAs found in blood plasma or serum can serve as non-invasive indicators for pancreatic cancer and urothelial carcinoma of the bladder [11, 12].

In CC, a new research report has pinpointed circSLC26A4 as a cancer-causing gene [13]. However, as far as we know, the role of circSLC26A4 in regulating the CC occurrence and development via exosomes remains unclear. The research investigated the possibility of using plasma exosomal circSLC26A4 as a liquid biopsy marker for CC. We compared exosomal circSLC26A4 expression derived from cervical normal epithelial and cancer cells. Additionally, we detected the exosomal circSLC26A4 expression in patients’ plasma and compared different cervical lesion levels in order to identify the significant of exosomal circSLC26A4 in the clinical diagnosis of CC.

## Material and methods

### Patients recruitment and samples collection

The patients diagnosed with CC (n = 18), high-grade squamous intraepithelial lesion (HSIL) (n = 18) and healthy volunteers (n = 20) were recruited from May 30, 2023, to August 1, 2023, from Zhengzhou University Affiliated Zhengzhou Central Hospital. The study was approved by the Institutional Research Ethics Committee of Zhengzhou Central Hospital (Ethical number: 202364). This trial was registered at the Chinese Clinical Trial Registry (www.chictr.org.cn, trial registration number ChiCTR2300072187, First registration time: 06/06/2023). Informed consent was obtained and signed from each patient before collecting. No patient had received any chemotherapy or radiotherapy before blood collection.

The clinical and pathological characteristics of the CC specimens are presented in Table1, including age, FIGO stage, lymphatic metastasis, intravascular cancerous embolus, and perineural invasion. Plasma was isolated from blood specimen, centrifuged at 2,000 ×g for 10 min then at 10,000 ×g for 10 at 4°C, and then stored at -80°C until exosome were extracted.

**Table 1:** Sequence primers designed for qRT-PCR.

	Forward	Reverse
circSLC26A	5-TCCAAGTGCTGGTCTCACAG-3	5-CCATATCCGACAGGAACTGC-3
has-miR-16-5p	5-TTTTAGCAGCACGTAAATATTGGCGA-3	-

### Cell culture

Cell lines (CaSki, SiHa, and HeLa) of CC and human cervical epithelial cells (HcerEpic) were purchased from the Chinese Academy of Sciences Cell Bank (Shanghai, China) and cultured in DMEM (Invitrogen) or RPMI-1640 medium (Invitrogen). The medium was supplemented with 10% fetal bovine serum (FBS) at 37°C in a humidified atmosphere with 5% CO_2_. The cell supernatants were collected separately for each cell type.

### Exosome isolation

The exosomes were isolated from the cell culture medium using the Total Exosome Isolation Kit (Beibei Biotechnology, Zhengzhou, China). The cell culture medium was filtered using a 0.22-μm filter. The cell culture medium samples were thawed in a 25°C-water bath and then centrifuged at 2,000 ×g for 30 min to remove cells and debris. Next, the cell culture medium was mixed with total exosome isolation reagent in a 4: 1 ratio and vortexed to obtain a homogeneous mixture. After 30 min of incubation at 4°C, the sample was centrifuged at 10,000 ×g for 10 min at room temperature. The exosome pellet was resuspended in phosphate-buffered saline (PBS).

The exosomes were isolated from plasma using the Total Exosome Isolation Kit (Beibei Biotechnology, Zhengzhou, China). Plasma samples were thawed on ice and centrifuged at 2,000 ×g for 30 min to remove blood cells and other impurities. Next, the plasma was mixed with total exosome isolation reagent in a 2:1 ratio and vortexed to obtain a homogeneous mixture. After 30 min of incubation at 4°C, the sample was centrifuged at 10,000 ×g for 10 min at room temperature. The exosome pellet was resuspended in PBS.

### Exosomes characterization

Exosomes were measured using the Bicinchoninic Acid (BCA) Protein Assay Kit (Solarbio, Beijing, China). Then, exosomes were stored at 4°C for transmission electron microscopy (TEM) and nanoparticle tracking analysis (NTA) and used within 48 h. Other exosomes were stored at -80°C until use.

The exosomal markers CD63, flotillin 2, and GAPDH were analyzed by western blot for different cell lines, exosomes derived from different cell lines, and exosomes derived from plasma exosomes. Additionally, NTA was used to measure exosome size distribution and zeta potential with the Zetasizer Nano S (Particle Metrix, Germany). Exosomes were fixed with 2% glutaraldehyde stationary liquid. Exosome suspension was dropped onto the copper grid with carbon film for 5 min, whereas 2% uranyl acetate was dropped on the copper grid to stain for 5 min at room temperature. The exosomes are observed using TEM (HITACHI, Japan).

### Western blot analysis

Exosomes lysate and cell lysate were prepared by RIPA Lysis Buffer (Yazyme, China) added with Protease Inhibitor Cocktail (Yazyme) and Phosphatase Inhibitor Cocktail (Yazyme). Total proteins were separated in SDS-polyacryla-mide gel (Yazyme) and transferred to polyvinylidene fluoride (PVDF) membranes (Beyotime). The membranes were blocked using Protein Free Rapid Blocking Buffer (Yazyme) for 15 min at room temperature. The blocked membranes were incubated overnight at 4°C with antibodies specific for the CD63 (Abcam, Cambridge, UK; 1:1000), flotillin 2 (Abcam, 1:1000), and GAPDH (Abcam, 1:1000). The membranes were washed three times with PBST and then incubated with HRP-conjugated secondary antibodies (Abcam, 1:5000) for 1 h. After the final wash with PBST, the proteins were detected using enhanced chemiluminescence (ECL) reagents (Pierce).

### Quantitative reserve transcriptase chain reaction (qRT‑PCR)

Total RNA was extracted from cellular and plasma exosome samples using a circRNA Isolation Kit (Beibei Biotechnology, Zhengzhou, China). H-miR-16 was used as an endogenous control for circSLC26A4 (Table 1). Then, cDNA was synthesized from total RNAs using a Reverse Transcriptase Kit (Beibei Biotechnology, Zhengzhou, China). The qRT-PCR was performed using LightCycler480 (Roche Applied Science, Basel, Switzerland) and qPCR Mix^SYBR Green^ I (Beibei Biotechnology, Zhengzhou, China). Cycling conditions: denaturation at 95 ◦C for 1 min for the first cycle, 94 ◦C for 30s thereafter, annealing at 60 ◦C for 30 s, and elongation at 72 ◦C for 30 s for 45 cycles. The relative plasma or cell supernatants exosomal circSLC26A4 levels were normalized against H-miR-16 using the 2^−ΔΔCt^ method.

### Statistical analysis

All statistical analyses were performed using SPSS 26.0 software (SPSS). Differences in the levels of cell or plasma exosomal circSLC26A4 between groups were determined by t-test. Associations of plasma exosomal circSLC26A4 with clinical parameters were examined by the Mann-Whitney. Establish the receiver operating characteristic (ROC) curves and the area under the curves (AUC) to evaluate the predictive value of plasma exosomal circSLC26A4 in CC. *P*-values < 0.05 were statistically significant for all tests.

## Results

### Exosomal circSLC26A4 was highly expressed in CC cell lines culture medium

**Fig 1:**
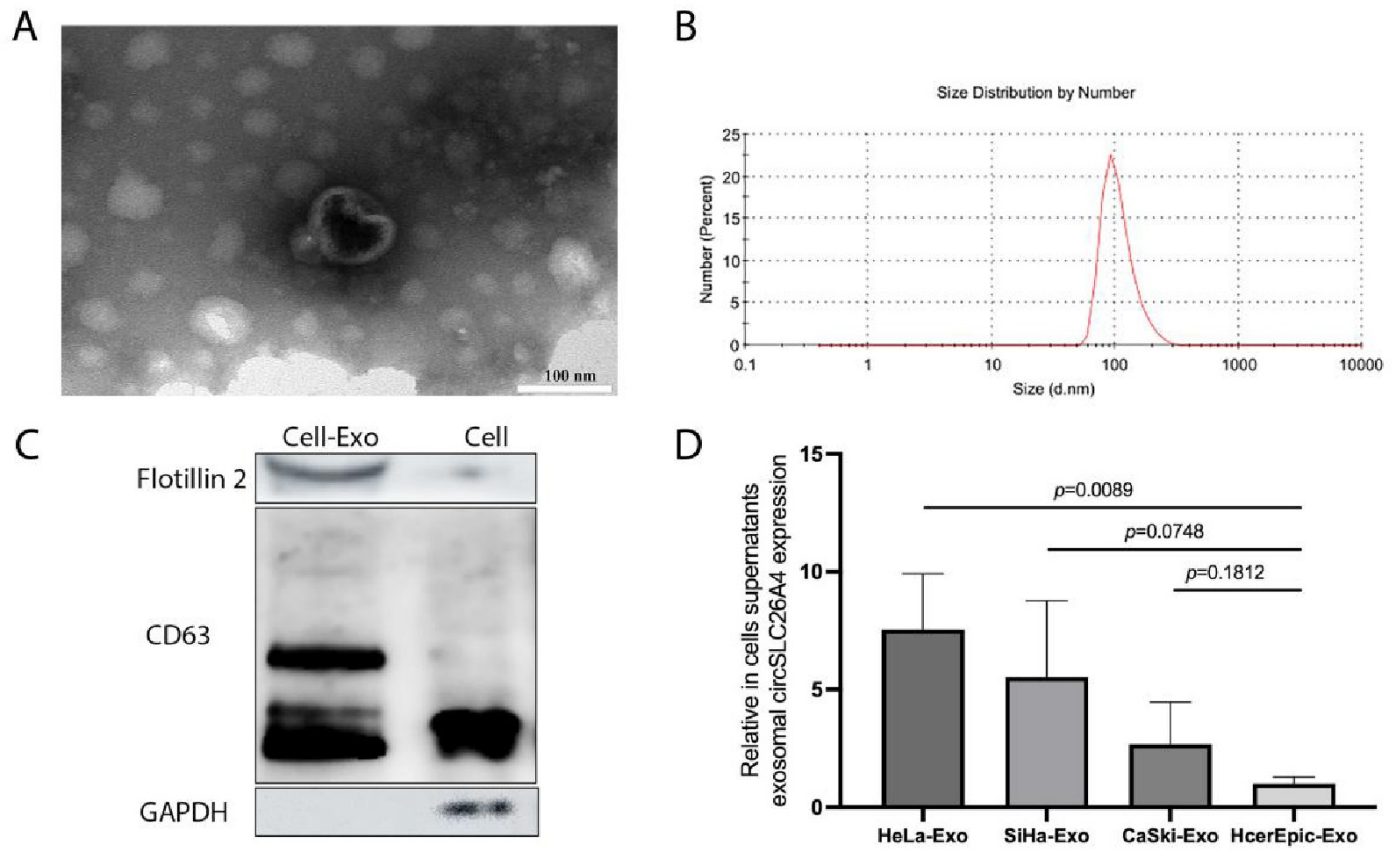
The circSLC26A4 is overexpressed in the exosome of CC cell lines culture medium. (A) Representative transmission electron microscopy (TEM) images of cell culture medium exosome. Scale bar, 100 nm. (B) Particle size distribution of cell culture medium exosome was measured using nanoparticle tracking analysis (NTA). (C) Protein immunoblots of exosomes, including the typical markers (flotillin 2, CD63). (D) RT-PCR assay was performed to measure the circSLC26A4 expression in different CC cell lines (SiHa, HeLa, and CaSki) and HcerEpic cell line medium-derived exosomes were described as a control. n = 12.

CC tissues and cells expressed high circSLC26A4 levels. We analyzed the exosomal circSLC26A4 expression in three human CC cell lines (CaSki, SiHa, and HeLa) and one normal cell line (HcerEpic) to investigate whether exosomal circSLC26A4 derived from CC cells is highly expressed. The exosomes derived from the culture medium were identified using TEM, NTA, and western blot (Fig 1A–1C). The circSLC26A4 was highly expressed in exosomes derived from CC cell lines than HcerEpic cells (HeLa: FC (fold change) = 7.55; SiHa: FC = 5.51; CaSki: FC = 2.68, HcerEpic: FC = 1.00; *P* = 0.270). Additionally, HeLa cell-derived exosomal circSLC26A4 expression was significantly higher than HcerEpic cell-derived expression (*P* = 0.0089). Consistent with our conjecture, the above results suggests that high-expressed exosomal circSLC26A4 was significantly associated with CC occurrence and could serve as a biomarker for CC.

### Plasma exosomal circSLC26A4 expression levels in patients with CC

**Fig 2:**
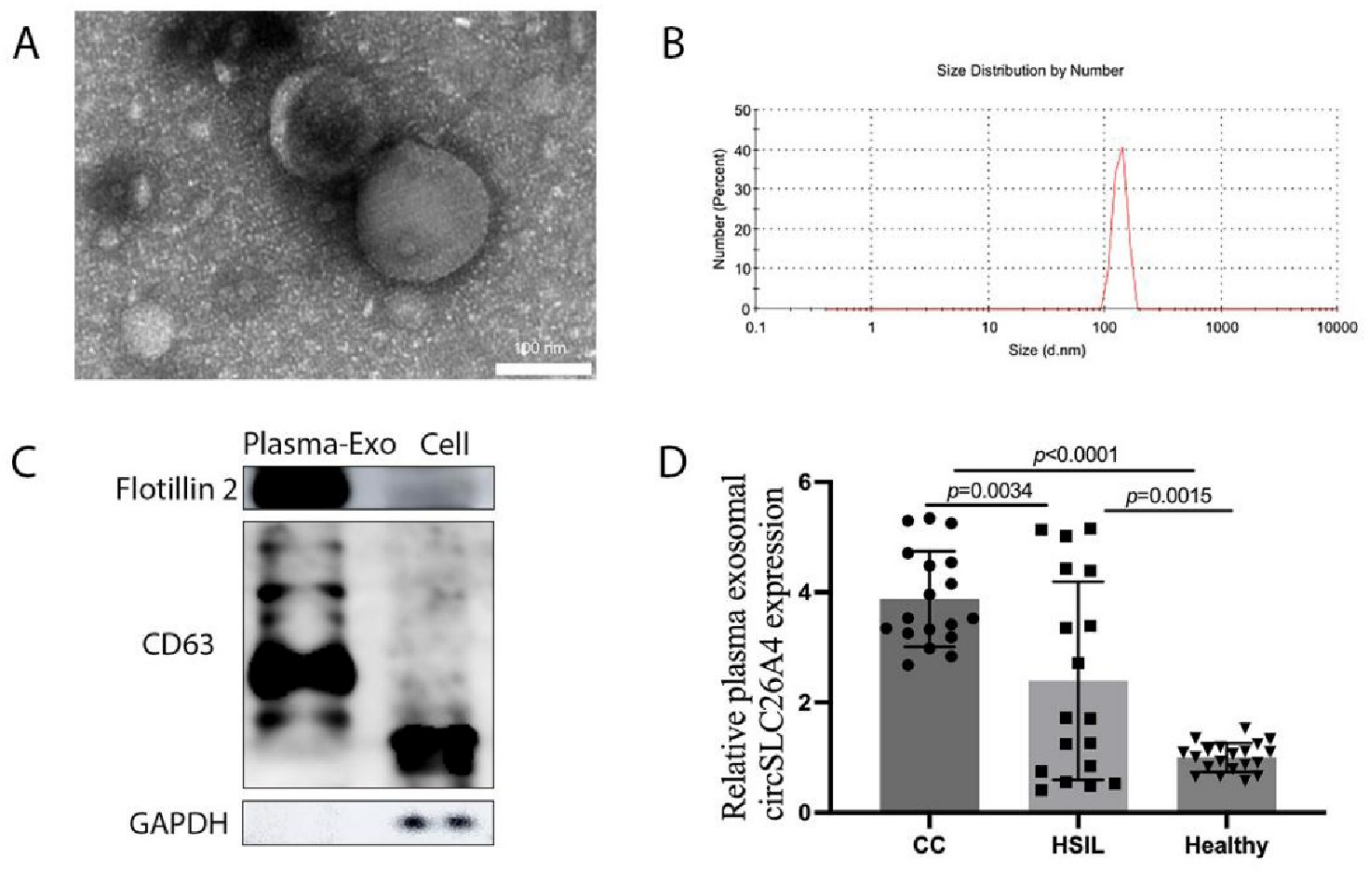
The circSLC26A4 is overexpressed in the plasma exosome of CC patients. (A) Representative transmission electron microscopy (TEM) images of plasma exosome. Scale bar, 100 nm. (B) Particle size distribution of plasma exosome was measured using nanoparticle tracking analysis (NTA). (C) Protein immunoblots of exosomes, including the typical markers (flotillin 2, CD63). (D) RT-PCR indicated the circSLC26A4 expression in CC and healthy plasma.

We collected blood samples from 18 CC patients, 18 cervical HSIL patients, and 20 healthy controls to determine whether circSLC26A4 can be used as a biomarker for detecting CC in plasma exosomes. Plasma-derived exosomal were detected through the use of TEM, NTA, and western blot assay of protein biomarkers (Fig 2). As anticipated, circSLC26A4 originating from plasma exosomal showed higher levels in CC patients compared to cervical HSIL patients and healthy controls (all *P* < 0.05). Moreover, exosomal circSLC26A4 was overexpressed in cervical HSIL patients than in healthy controls (*P* < 0.05). The above results demonstrate that the exosomal circSLC26A4 derived from plasma is a promising biomarker for detecting CC.

### Diagnostic values of plasma exosomal circSLC26A4 for CC

**Fig 3:**
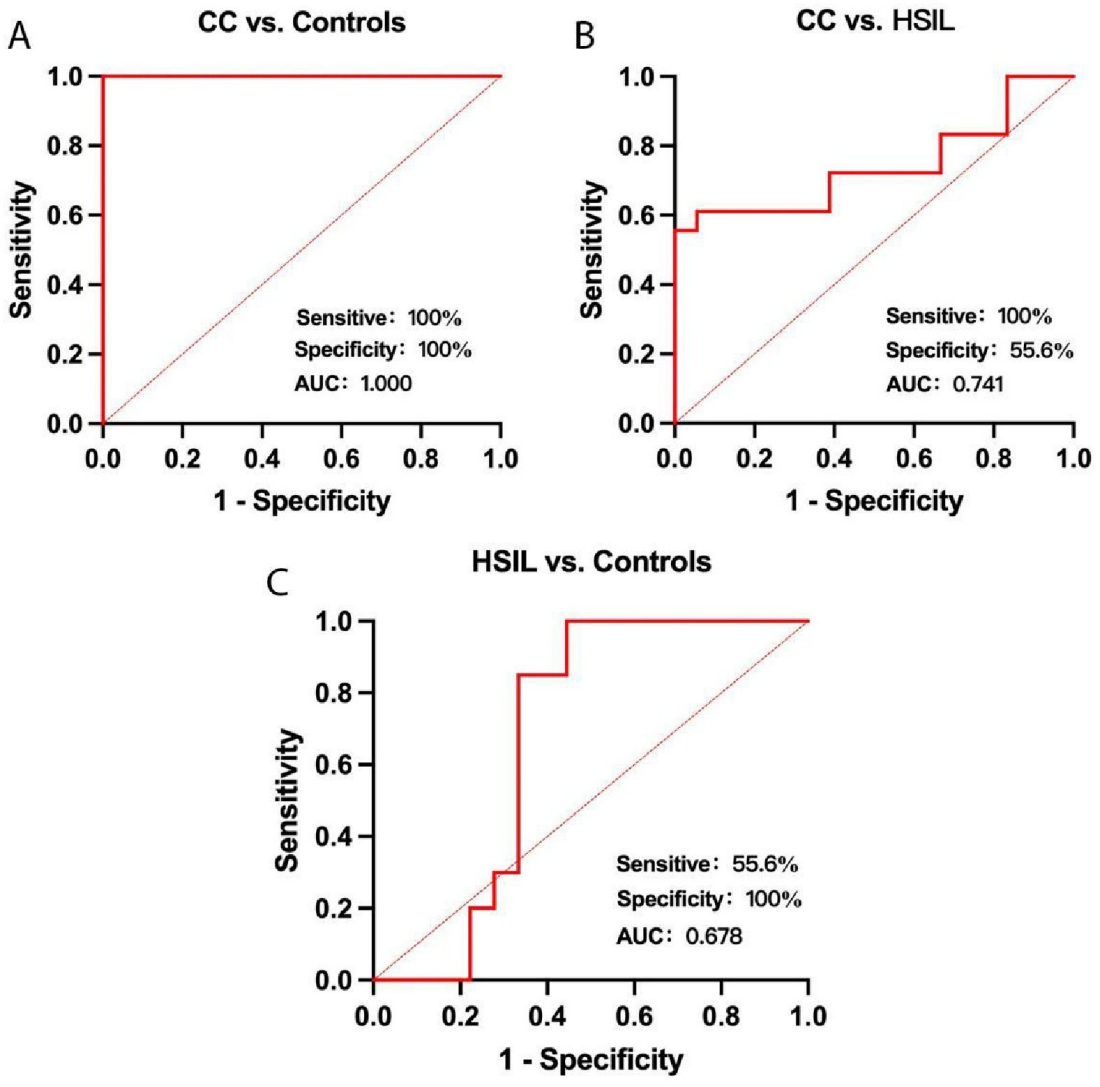
Diagnostic value of ROC curve analysis of plasma exosomal circSLC26A4. Diagnostic value of the plasma exosomal circSLC26A4 in differentiating CC patients from controls (A), CC and HSIL patients (B), and HSIL patients and controls(C).

Roc analysis was conducted to assess the diagnostic precision of plasma exosomal circSLC26A4 as a potential marker for CC tumors. Plasma exosomal circSLC26A4 was able to differentiate between CC patients and normal controls with high accuracy, achieving an AUC of 1.000 (95% CI: 1.000–1.000; specificity: 100%; sensitivity: 100%; Youden Index: > 2.103; Fig 3A). The AUC value of plasma exosomal circSLC26A4 for differentiating CC from HSIL was 0.741 (95% CI: 0.569–0.913; specificity: 55.6%; sensitivity: 100%; Youden Index: > 2.202; Fig 3B). The AUC value of plasma exosomal circSLC26A4 for differentiating HSIL patients and normal controls was 0.678 (95% CI: 0.4768–0.8787; specificity 100%; sensitivity 55.6%; Youden Index: > 1.619; Fig 3C).

### Relationship between plasma exosomal circSLC26A4 expression and clinical variables in patients with CC

We conducted Mann-Whitney tests to determine if there is a relationship between the amount of plasma exosomal circSLC26A4 and the clinical factors of CC patients, including age, FIGO stage, intravascular cancerous embolus, perineural invasion and lymph node metastasis. Lymph node metastasis and FIGO stage was positively correlated with plasma exosomal circSLC26A4 expression (*P* < 0.05). Nevertheless, there was no significantly association between plasma exosomal circSLC26A4 expression and age, intravascular cancerous embolus, and perineural invasion (*P* > 0.05; Table 2).

**Table 2:** Association between CC patients clinicopathological Factors and plasma exosomal circSLC26A4 expression.

Characteristic	Total	circSLC26A4 expression	*Z*	*P*-value
Total number of CC patients	18	-	-	-
**Age**				
< 55	10	3.47 (3.20, 4.54)	-0.444	0.657
≥ 55	8	3.84 (3.20, 5.07)		
**FIGO Stage**				
I-II	12	3.33 (3.03, 4.41)	-1.967	0.049
III-IV	6	4.32 (3.85, 4.74)		
**Lymphatic metastasis**				
Positive	5	4.15 (3.74, 4.51)	-3.000	0.003
Negative	9	3.26 (2.91, 3.38)		
undetected	4	-		
**Intravascular cancerous embolus**				
Positive	8	3.30 (2.92, 3.50)	-0.849	0.396
Negative	4	3.43 (3.07, 4.41)		
undetected	6	-		
**Perineural invasion**				
Positive	3	3.33 (3.26, 3.43)	-0.092	0.926
Negative	9	3.34 (2.98, 3.53)		
undetected	6	-		

## Discussion

Growing proof indicates that exosomal circRNAs have a significant impact on cancer development in various types of human tumor, including hepatocellular carcinoma [14, 15], bladder cancer [16], non-small cell lung carcinoma (NSCLC) [17], and gastric cancer [18, 19]. CircRNAs are distinguished by their covalently closed loop structures that lack 5′ caps and 3′ poly tails in contrast to other non-coding RNAs. Moreover. the stable structure of circRNAs is resistant to environmental degradation [20–22]. Therefore, the molecular structure of circRNAs is advantageous for clinical applications as a biomarker.

Current studies are centered on investigating the expression patterns and roles of circRNAs in various human diseases, with a particular emphasis on cancers. Numerous circRNAs in CC have been identified as tumorigenic [23, 24]. An example of this is circNEIL3, which acts a ceRNA by competitively binding to miR-137, leading to the indirect increase of KLF12 expression and the stimulation of proliferation CC cells [25]. An additional instance involves circular RNA 0001823 exacerbating the proliferation and spread of CC cells through modulation of the microRNA-613/RAB8A axis [26]. A recent study showed that circSLC26A4 is abundantly present in different types of cancers and is strongly linked to tumors initiation, progression, and unfavorable outcomes for cancer patients. In NSCLC, circSLC26A4 expression has increased and could enhance cancer cell growth by inhibiting miR‑15a maturation [27]. The circSLC26A4 was strongly associated with poor patient survival in CC and promotes CC progression by upregulating HOXA7 via miR-1287-5p adsorption [13]. Additionally, the advantages of exosomal circRNA detection as a liquid biopsy technique in cancer diagnosis were significant, and there are currently few relevant studies in CC. Research findings show that circRNA_PVT1 triggers EMT in CC cells by interacting with miR-1286 through the exosome pathway [28]. Since the regulatory function of exosomal circSLC26A4 in CC is not yet clear and exosomal circRNA could be useful in diagnosing CC, we conducted a study to investigate the clinical potential of exosomal circSLC26A4 derived from CC.

Our study discovered that exosomal circSLC26A4 derived from CC cell lines was significantly upregulated compared to normal cell lines (HcerEpic). Exosomal circSLC26A4 expression was highest in the HeLa cell medium. Given that HeLa cells are derived from cervical adenocarcinoma, we speculate that the exosome circSLC26A4 may be closely involved in the occurrence and development of adenocarcinoma. However, this hypothesis needs further research to explore and prove. We collected plasma exosomes from 56 volunteers, including 18 CC patients, 18 HSIL, and 20 healthy women, and quantified the plasma exosomal circSLC26A4 levels further to clarify the application of exosomal circSLC26A4 in CC. Plasma exosomal circSLC26A4 levels were significant elevated in CC patients compared to the other groups, as shown by the results. Moreover, plasma exosomal circSLC26A4 proved to be a reliable indicator for tracking treatment progress in patients with CC. We also conducted a statistical analysis of the relationship between the plasma exosomal circSLC26A4 expression level and clinical information of the CC patients. The findings indicate a positive association between plasma exosomal circSLC26A4 levels and lymph node metastasis, which aligns with the CaSki exosomal circSLC26A4 expression in CC cell lines. Nonetheless, the plasma exosomal circSLC26A4 expression level did not show a notable connection with the patient’s age, intravascular cancerous embolus, and perineural invasion in terms of clinical features. The size of our study sample may be the reason for this correlation. In conclusion, the findings demonstrate that exosomal circSLC26A4 can function as a biomarker for non-invasive liquid biopsy non-invasive screening of CC.

However, our research still has some limitations. Initially, the small sample size may result in the clinical prediction of plasma exosomal circSLC26A4 for cervical cancer (as shown in Fig 3) being adapted to the random noise in the data, making it unable to be generalized to a wider population and failing to reflect the overall situation. This will affect the sensitivity and specificity of plasma exosomal circSLC26A4 in diagnosing cervical cancer. The specificity and sensitivity of plasma exosomal circSLC26A4 for the CC group and the control group both reach 100%, which may indicate overfitting problems and lead to a large generalization error of the model. Hence, in order to enhance the clinical application value of plasma exosomal circSLC26A4 in cervical cancer, a larger sample size study is needed. Furthermore, when using circSLC26A4 to predict cervical cancer, it is necessary to fully combine existing mature tumor markers and clinical pathological factors to ensure accurate prediction of cervical cancer.

## Conclusion

The initial study shows that high levels of plasma exosomal circSLC26A4 can be used to identify patients with CC. To sum up, plasma exosomal circSLC26A4 could be a valuable indicator for the non-invasive detection of CC.

## Supporting information

S1 Raw image (PDF)

S1 Data (XLS)

S2 Data (XLS)
