# Supplementary figures and images for: Identification of exosomal circSLC26A4 as a liquid biopsy marker for cervical cancer
Source: PLoS One. 2024 Jun 11;19(6):e0305050. doi: 10.1371/journal.pone.0305050 (PMC11166277; doi:10.1371/journal.pone.0305050)

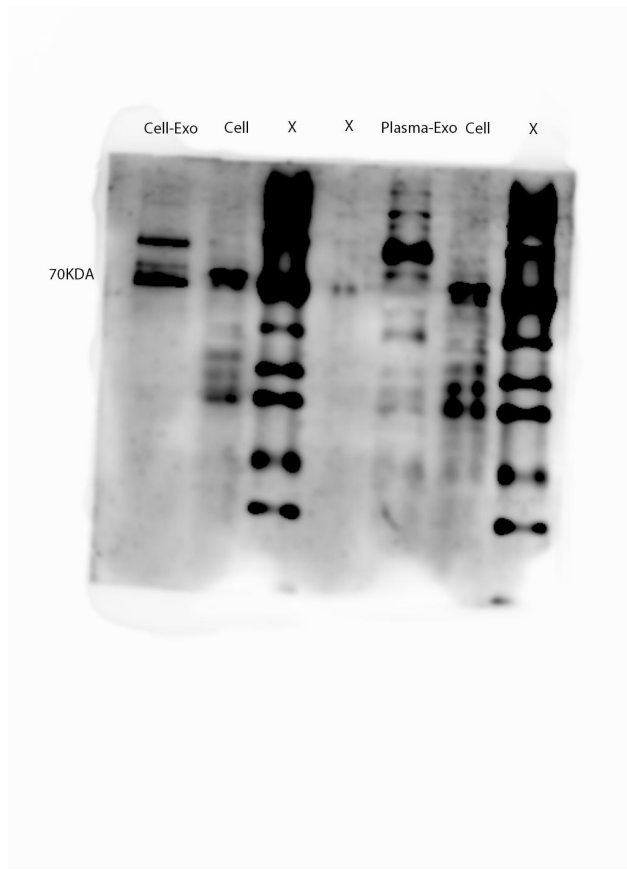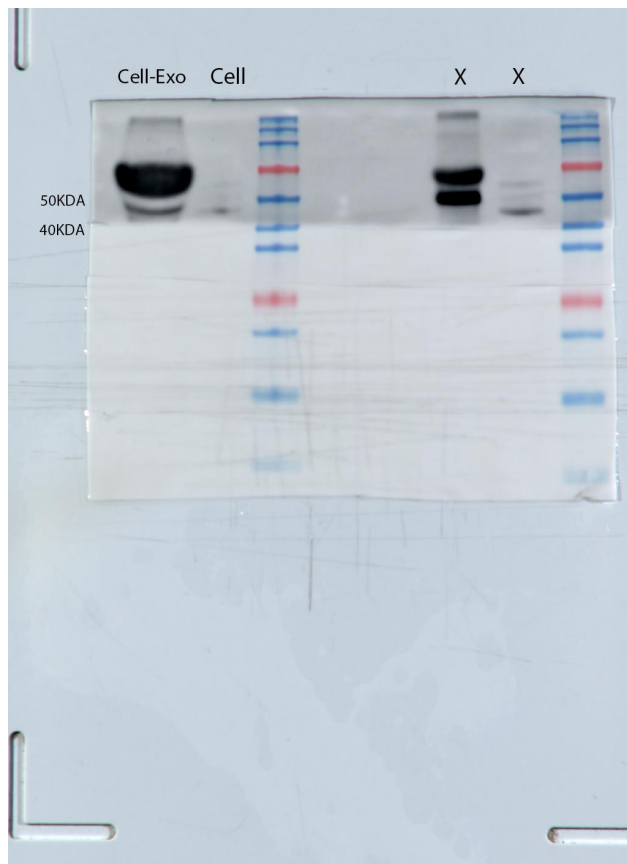

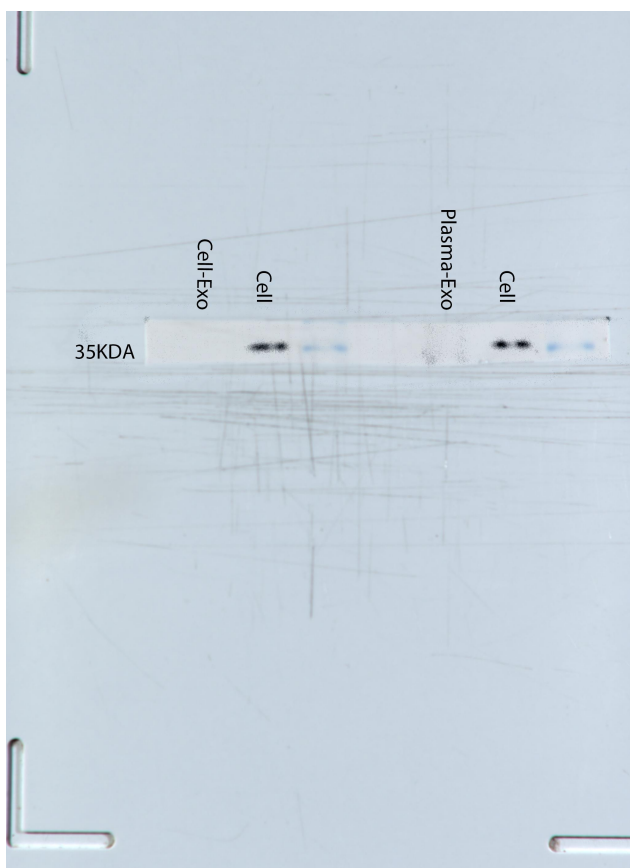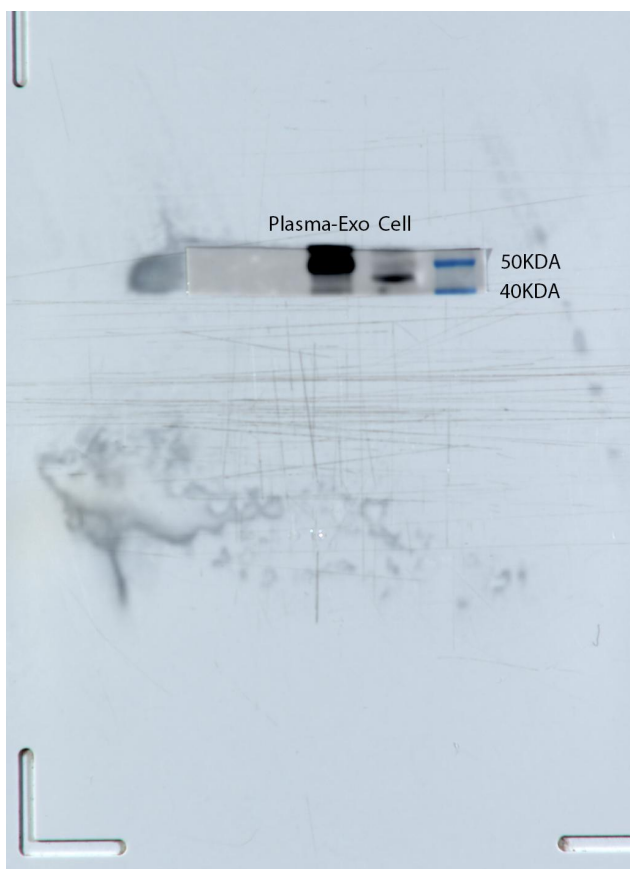

Supplement: S1 Raw image — (PDF) [file pone.0305050.s001.pdf]
